# Absence of Erythrocyte Sequestration and Lack of Multicopy Gene Family Expression in *Plasmodium falciparum* from a Splenectomized Malaria Patient

**DOI:** 10.1371/journal.pone.0007459

**Published:** 2009-10-14

**Authors:** Anna Bachmann, Claudia Esser, Michaela Petter, Sabine Predehl, Vera von Kalckreuth, Stefan Schmiedel, Iris Bruchhaus, Egbert Tannich

**Affiliations:** 1 Bernhard Nocht Institute for Tropical Medicine, Hamburg, Germany; 2 Department of Tropical Medicine, University Medical Center Hamburg-Eppendorf, Hamburg, Germany; Bernhard Nocht Institute for Tropical Medicine, Germany

## Abstract

**Background:**

To avoid spleen-dependent killing mechanisms parasite-infected erythrocytes (IE) of *Plasmodium falciparum* malaria patients have the capacity to bind to endothelial receptors. This binding also known as sequestration, is mediated by parasite proteins, which are targeted to the erythrocyte surface. Candidate proteins are those encoded by *P. falciparum* multicopy gene families, such as *var*, *rif*, *stevor* or *PfMC-2TM*. However, a direct *in vivo* proof of IE sequestration and expression of multicopy gene families is still lacking. Here, we report on the analysis of IE from a black African immigrant, who received the diagnosis of a malignant lymphoproliferative disorder and subsequently underwent splenectomy. Three weeks after surgery, the patient experienced clinical *falciparum* malaria with high parasitemia and circulating developmental parasite stages usually sequestered to the vascular endothelium such as late trophozoites, schizonts or immature gametocytes.

**Methodology/Principal Findings:**

Initially, when isolated from the patient, the infected erythrocytes were incapable to bind to various endothelial receptors *in vitro*. Moreover, the parasites failed to express the multicopy gene families *var*, A-type *rif* and *stevor* but expression of B-type *rif* and *PfMC-2TM* genes were detected. In the course of *in vitro* cultivation, the parasites started to express all investigated multicopy gene families and concomitantly developed the ability to adhere to endothelial receptors such as CD36 and ICAM-1, respectively.

**Conclusion/Significance:**

This case strongly supports the hypothesis that parasite surface proteins such as PfEMP1, A-type RIFIN or STEVOR are involved in interactions of infected erythrocytes with endothelial receptors mediating sequestration of mature asexual and immature sexual stages of *P. falciparum*. In contrast, multicopy gene families coding for B-type RIFIN and PfMC-2TM proteins may not be involved in sequestration, as these genes were transcribed in infected but not sequestered erythrocytes.

## Introduction

The most severe form of malaria is caused by the protozoan parasite *Plasmodium falciparum* and is one of the major public health problems with over two million deaths worldwide. Virulence of *P. falciparum* has been linked to the ability of infected erythrocytes (IE) to adhere to a range of endothelial cell surface receptors expressed on blood vessel walls. This phenomenon known as sequestration allows the parasites to avoid spleen-dependent killing mechanisms [1]. The spleen removes erythrocytes that are less deformable, such as parasite-infected cells [2], and those sensitized by IgG [3] during acute *falciparum* malaria. In addition to the removal of entire red blood cells, the spleen is able to extract selectively parasites from the erythrocytes but leaving the remaining cells within the circulation. This mechanism also known as “pitting” appears to be particularly relevant for the removal of dead parasites following anti malaria treatment [4], [5].

Membrane proteins that mediate binding to endothelial cells are exposed to the host's immune system. To avoid immune recognition and subsequent killing of IE these surface antigens are consistently changed by means of antigenic variation. Therefore, parasites are equipped with several gene families that encode variant antigens displayed on the erythrocyte surface. The best-characterized multicopy gene family of *P. falciparum* is the *var* gene family, which codes for the high-molecular weight *P. falciparum* erythrocyte membrane protein-1 (PfEMP-1). Proteins of this family have been shown to be linked to cytoadhesion of IE to different endothelial receptors such as CD36, ICAM-1 (intercellular adhesion molecule-1), VCAM-1 (vascular cell adhesion molecule-1) and P-selectin [6]–[10]. PfEMP-1 proteins undergo antigenic variation by switching *var* gene expression of a repertoire of 60 genes per haploid genome. *Var* genes have been subgrouped according to their upstream region, chromosomal localization and orientation into the three major groups A, B and C [11], [12].

Apart from *var* genes several other multicopy gene families were characterized in asexual *P. falciparum* stages. These include r*if* (repetitive interspersed family), *stevor* (subtelomeric variable open reading frame) and *Pfmc-2TM* (*P. falciparum* maurer's clefts 2 transmembrane) genes. They code for exported proteins with a predicted two-transmembrane topology and an intermediate hypervariable loop, which is assumed to be surface exposed [13]–[15]. RIFIN proteins were divided into A- and B-type RIFINs. A-type RIFIN proteins are associated with the Maurer's clefts (MC), a membranous network which is involved in the export of proteins from the parasitic cytosol to the IE surface, whereas B-type RIFIN proteins appear to be restricted to the parasitic cytosol [16], [17]. STEVOR proteins are associated with the MC and were recently found to be surface exposed [18]–[21]. Members of the PfMC-2TM protein family also show an association with the MC and are located at protrusions of the IE membrane known as knobs, which represent contact points of the IE with endothelial cells [18]–[20]. In addition, genes of the s*tevor* and *Pfmc-2TM* families have switching rates similar to that of the *var* gene family, at least in one laboratory strain [14], suggesting an involvement of these protein families in antigenic variation. Nevertheless, the surface exposure and topology of RIFIN, STEVOR and PfMC-2TM proteins remains controversial and the biological function is as yet unknown.

Here we report a case of *P. falciparum* malaria in a splenectomized patient whose parasites were non-sequestered to investigate the link between expression of multicopy gene families and adherence of IE to endothelial receptors.

## Materials and Methods

### Sample preparations and in vitro culture

For *in vitro* cultivation approximately 1 mL of residual blood was obtained from the diagnostic department of the Bernhard Nocht Institute for Tropical Medicine, Hamburg, immediately after the diagnosis of *P. falciparum* malaria with 24% parasitemia has been reported and informed consent was obtained from the patient. Erythrocytes were isolated by ficoll gradient centrifugation. An aliquot of 200 µL was separated (*ex vivo* sample) and the remaining parasites were subsequently cultivated in 0+ erythrocytes using a protocol adopted from Trager and Jensen [22]. In the following, ring stage parasites were harvested approximately every week after performing synchronization using standard methods [23] for analyzing the expression of *var* genes. Additionally, mixed stage parasites as those seen in the *ex vivo* sample were harvested on day 29, 38 and 44 of subsequent cultivation to investigate the expression of *rif*, *stevor* and *Pfmc-2TM* genes.

### DNA purification and genotyping

To determine the number of *P. falciparum* genotypes in the *ex vivo* blood sample in subsequent cultures, MSP-1-PCR was performed. For this, genomic DNA was isolated (QIAamp DNA Mini Kit, Qiagen) and genotyping was carried out as described elsewhere [24].

### RNA purification, cDNA synthesis and PCR

Isolation of RNA was conducted as previously described [25]. The SuperScript III Kit (Invitrogen) was used to generate cDNA. For analyzing *var* gene expression the reaction was primed with a mixture of two degenerate ATS-specific reverse primers, namely var2 [26] and var4 (5′-GTW CCA AAW ART TCA TTT TCT TTW CKT TTW ABT TCA TCA TAT ATA TC-3′). First strand cDNA synthesis for analyzing the expression of *rif*, *stevor* and *Pfmc-2TM* gene families was performed using random hexamers. In parallel, a negative control with an equal amount of corresponding RNA samples was prepared in which the reverse transcriptase was omitted. For the various PCR reactions targeting the different multicopy gene families the following pairs of primers were used: *var* group A, B and C (upsA1_for, 17deg_for, RT-5B1.1_for and DBL_FADall-rev) [27], *rif-A and -B* ([16], *stevor* (for: 5′-TCC RCA TTA TCA YAA TG-3′ and rev: 5′-AAA TGT TTC TTG CAT TCA TGT TTC-3′) and *Pfmc-2TM* (for: 5′-TGG AAT AAT ATT KGG ATT -3′ and rev: 5′-TCA GGA AAR WAA CGA RC-3′). In virtual PCR experiments (http://insilico.ehu.es) the various primer pairs amplified 60% and 46%, respectively, of the *var* groups A and C genes, 97% and 90%, respectively, of the *rif-A* and *-B* families, 72% of the *stevor* and 83% of the *Pfmc-2TM* gene repertoire in the 3D7 genome using the 1 mismatch configuration. The *var* group B primer showed no result in the *in silico* amplification. However, unbiased amplification was confirmed by cloning and subsequent sequencing of cloned the PCR products using genomic DNA from 3D7 as well as from the isolate of the splenectomized patient as a templates (supporting information). A PCR for the constitutively expressed housekeeping gene *seryl*-tRNA synthetase was used as positive control for gene expression [28]. To control PCR performance, PCR reactions were conducted using genomic DNA of the isolate as template.

### Cloning, sequencing and analysis of PCR products

PCR products of the *ex vivo* samples corresponding to *rif* group B and *Pfmc-2TM* were purified by gel extraction using the NucleoSpin Extract II Kit (Macherey-Nagel), cloned into the pCR2.1 TOPO vector and transformed into TOP10 cells (TOPO TA Cloning Kit, Invitrogen). Sequencing was done by AGOWA GmbH (Berlin) and analysis was performed using the MacVector version 9.5.2 software.

### Static cytoadhesion assay

For static cytoadhesion assays Chinese hamster ovary cells defective in glycosaminoglycan biosynthesis (CHO-745, American Type Culture Collection) were transfected using Lipofectamine 2000 (Invitrogen) complexed with pAcGFP1-N1 (BD Biosciences Clontech), pAcGFP1-N1-*cd36*, pAcGFP1-N1-*icam-1*, pAcGFP1-N1-*p-selectin* or pEGFP-*vcam1*. *Cd36* and *icam-1* cDNAs were kindly provided by Mo Klinkert (Bernhard Nocht Institute for Tropical Medicine, Hamburg), *p-selectin* cDNA by Rodger McEver (Oklahoma Medical Research Foundation, Oklahoma City) and pEGFP-*vcam1* by Francisco Sanchez-Madrid (Hospital Universitario de la Princesa, Madrid). Stable transfectants were selected by growth in Ham's F-12 medium (PAA Laboratories GmbH) supplemented with 10% fetal calf serum, penicillin-streptomycin and G418 (0.7 mg/mL). CHO-745 cells expressing the endothelial receptors CD36, ICAM-1, VCAM-1 and P-selectin, and CHO-745 control cells expressing GFP were plated onto 15-mm coverslips and maintained in 24-well plates. The cells were seeded at a density of 24,000 cells per well and grown for 24 hours. Culture medium was replaced with 0.5 mL IE of trophozoite/schizont parasite stages in binding medium (RPMI medium 1640 supplemented with 2% glucose, pH 7.2) at 1% hematocrit. Parasitemia was 15% on day 1, 2.8% on day 8, 4,0% on day 15, 5.5% on day 30, 5.6 on day 39 and 3.8% on day 45. Incubation with IE was conducted for 1 hour at 37°C with gentle mixing at 10 minute intervals. Afterwards, unbound erythrocytes were gently removed by washing with binding medium and the cells were fixed with 1% glutaraldehyde in PBS for 1 hour at room temperature. Cells on coverslips were stained with Giemsa, and the number of adherent IE per 500 CHO-745 cells was determined using a light microscope. All assays were performed in triplicates.

## Results

### Case history

**Figure 1:**
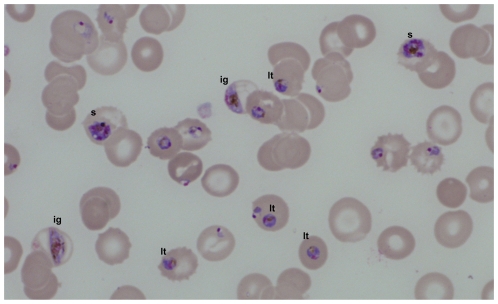
Plasmodia in peripheral blood of splenectomized patient. Shown are Giemsa-stained thin blood films containing various parasite stages, including those usually not seen in peripheral blood of *P. falciparum* malaria, such as late trophozoites (lt), schizonts (s) or immature gametocytes (ig).

Three weeks after immigrating to Europe, a 51-year-old woman from Cameroon attended a consultation at a hospital in Germany due to fever, asthenia and abdominal pain (Tab. 1). The physical examination revealed paleness, an enlarged spleen and pressure pain in the upper abdomen. Laboratory investigations showed reduced hemoglobin level of 100 g/L, pancytopenia, a high level of serum IgM as well as an increased polyclonal hyper-γ-globulin level. It is worth noting that malaria thick and thin blood films, as well as malaria antigen tests were repeatedly negative. However, serological malaria testing revealed high titers of antibodies to *P. falciparum* antigen. A CT-scan of the abdomen indicated an extensive splenomegaly with some focal calcifications and subcapsular hemorrhages as well as multiple enlarged abdominal lymph nodes. Accordingly, a malignant lymphoproliferative disorder was suspected and splenectomy was performed. The removed organ possessed a weight of 1.18 kg and was 16×15×8.5 cm in size. Histopathology showed increased numbers of plasma cells and massive deposition of malaria pigment (hemozoin). Three weeks after surgery the patient suffered from persistent fever and thrombocytopenia and the general condition worsened, which prompted the transfer to a hospital specialized in tropical medicine and infectious diseases. Laboratory at admission showed anemia with hemoglobin level of 79 g/L, and an increased creatinine serum level of 21 mg/L. In contrast to previous examinations, thin blood films now showed *P. falciparum*-infection with a high parasitemia of 24% and the presence of large quantities of parasite stages usually not seen in peripheral blood of *P. falciparum* malaria patients, such as late trophozoites, schizonts and immature gametocytes (Fig. 1). Consequently, diagnosis had to be revised to a hyperreactive malarial splenomegaly (HMS). Malaria therapy with chinine and doxycycline was initiated accordingly and the patient recovered rapidly, despite a markedly prolonged clearance of IE from the blood circulation (14 days). For the outpatient follow-up examinations the patient presented in a good medical condition.

**Table 1:** Demographic and clinical characteristics of the splenectomized patient.

Characteristic	
Country of origin	Cameroon
Time from patient's arrival in Germany to splenomegaly diagnosis	3 weeks
Time from splenomegaly diagnosis to splenectomy	6 weeks
Time from splenectomy to clinical malaria	3 weeks
Symptoms before splenectomy	Asthenia, weight loss, abdominal pain
Size and weight of spleen	16×15×8.5 cm (1.18 kg)
Serum IgM level before splenectomy^a^	21.3 g/L
Serum titer of antibodies against *Plasmodium falciparum* before splenectomy^b^	>1∶5,000
Blood tested for malaria before splenectomy^c^	Negative finding
Hypersplenism	Yes, moderate pancytopenia

a Normal serum IgM level, <2.3 g/L.

b Results from immunofluorescence test normal value, <1∶100.

c Several thin and thick blood smears and Plasmodium antigen test.

### Expression of P. falciparum multicopy gene families

**Figure 2:**
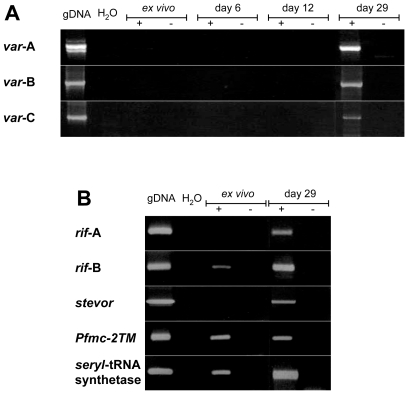
Multicopy gene family expression of IE during parasite culture. (A) Shown are ethidium bromide stained agarose gels of v*ar* RT-PCR products of RNA extracted from the *ex vivo* blood sample as well as from samples of subsequent parasite cultures at time points as indicated. Genomic parasite DNA (gDNA) and water (H_2_O), respectively, served as positive and negative controls. To rule out amplification of DNA that might have contaminated the various RNA preparations, all RNA samples were subjected to PCR with (+) and without (−) reverse transcription. (B) RT-PCR products from the multicopy gene families *rif*, *stevor*, *Pfmc-2TM* and from the housekeeping gene *seryl*-tRNA synthetase from *ex vivo* parasites and at day 29 of cultivation.

To examine whether the lack of sequestration of IE might be due to inadequate expression of *P. falciparum* multicopy gene families, RNA expression of *var*, *rif*, *stevor* and *Pfmc-2TM*, respectively, was analyzed by reverse transcription PCR in the *ex vivo* blood sample of the patient prior to anti-malaria treatment. The results indicated lack of transcription for the various *var* gene groups as well as for A-type *rif* and *stevor* genes (Fig. 2). In contrast, RNA was detected for B-type *rif* and *Pfmc-2TM* multicopy gene families as well as for the housekeeping gene *seryl*-tRNA synthetase (Fig. 2B). *Ex vivo* PCR products of B-type *rif* and *Pfmc-2TM* genes were subjected to DNA sequencing. Overall six diverse B-type *rif* and four *Pfmc-2TM* sequences were identified, none of which displayed high similarities to the conserved pseudogenes of 3D7 [17], [18]. In addition to the *ex vivo* analysis, parasites were taken into culture and *var* gene expression was analyzed on day 6 and 12 and for all multicopy gene families on day 29, 38 and 44 of cultivation. As in the *ex vivo* blood sample taken directly from the patient, no *var* RNA was detected on day 6 or day 12 of parasite cultivation (Fig. 2A). However, all multicopy gene families were expressed on day 29 (Fig. 2B) as well as at the following time points investigated (data not shown).

### Binding of infected erythrocytes to endothelial receptors

**Figure 3:**
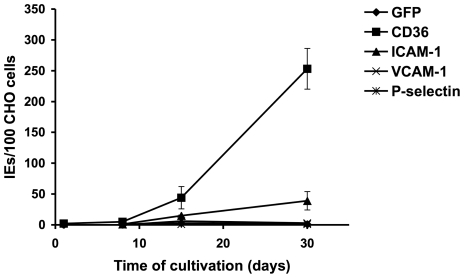
Binding of IE to common endothelial receptors during parasite culture. IE obtained *ex vivo* and from the time points day 8, day 15 and day 30 of subsequent parasite cultures were incubated with CHO-745 cells expressing endothelial receptors CD36, ICAM-1, VCAM-1 and P-selectin, respectively. CHO-745 cells expressing GFP were used as negative control. Bound erythrocytes were stained with Giemsa and the number of adherent IE per 100 CHO-745 cells is shown. Assays were performed in triplicates. All data are mean values (± standard deviation).

In parallel to RNA analyses, adherence of IE to the endothelial receptors CD36, ICAM-1, VCAM-1 and P-selectin, respectively, was analyzed by a static cytoadhesion assay using receptor-expressing CHO-745 cell lines. CHO-745 cells expressing GFP were used as controls. The results indicated lack of binding of IE to any of the four receptors during the first eight days of cultivation. However, on the two following time points (day 15 and 30) a gradual increase of IE binding was observed to CD36 and ICAM-1, which reached a maximum on day 30 of cultivation (Fig. 3) and did not further change during the following weeks (data not shown). At none of the time points investigated binding of IE to VCAM-1 or P-selectin was observed.

### MSP-1 genotyping

To determine the number of parasite genotypes initially present in the patient and subsequently during culture, MSP-1 genotyping was performed. The results indicated two different *P. falciparum* genotypes (K1-159 bp and Mad20-212 bp) in a ratio of approximately 3∶1 initially present in the patient. However, during culture the more abundant genotype outgrew and after 5 weeks of cultivation only genotype K1 was detected (data not shown).

## Discussion

Hyperreactive malarial splenomegaly (HMS) occurs mostly in long-term residents of areas where malaria is endemic [29]. Accordingly, HMS should be suspected in any patient originating from areas of malaria endemicity and displaying symptomatic or asymptomatic splenomegaly [30]. On the other hand, HMS is rarely seen in Western countries and some aspects of the disease show similarities to malignant lymphoproliferative disorder such as the extraordinary enlargement of the spleen, T lymphoproliferation or high level of serum IgM [30]–[34]. Moreover, HMS patients, although chronically infected with *Plasmodia*, may have a very low parasitemia, in many cases below the detection limit of thick blood film microscopy or even PCR [30]. Thus, misdiagnosis of HMS in patients from malaria endemic countries with subsequent splenectomy has been repeatedly reported from Western countries [30], [35]–[37]. The case presented here follows a similar pattern. The patient received the diagnosis of a lymphoproliferative disorder and was subjected to splenectomy, in particular, as thick blood films and malaria antigen tests were repeatedly negative. As in other cases of HMS following splenectomy, the patient developed acute *P. falciparum* malaria with high parasitemia of 24%[38]–[40]. In addition, and similar to other asplenic malaria patients, blood film microscopy revealed the presence of usually sequestered late asexual and early sexual parasite stages as well as a prolonged clearance of parasites following anti-malaria treatment [30], [39], [41]. These results strongly support the role of the spleen not only in controlling parasite burden but also in removing particular developmental parasite stages or dead parasites.

Two hypotheses for the appearance of mature parasites in the peripheral blood in splenectomized individuals have been proposed [38]. Firstly, it has been repeatedly postulated that the spleen modulates sequestration of IE although no underlying mechanism has been identified [40], [42]–[44]. The second assumption proposes that mature parasites, which for any reason do not sequester are readily removed from the circulation by a functioning spleen, which identifies them through their surface molecules or rheological properties. Thus, mature asexual and immature sexual parasites in splenectomized individuals would represent the sub-population of IE that failed to sequester in non-splenic tissues, presumably because they are not expressing appropriate ligands for cytoadherence [1]. This latter explanation would be fully consistent with our finding that the *ex vivo* parasites from the splenectomized patient do not express genes coding for proteins that are targeted to the erythrocyte surface and consequently these IE do not bind to common endothelial receptors as shown by our *in vitro* analyses. Interestingly, the *ex vivo*, non-binding parasites failed to express *var*, A-type *rif* and *stevor* genes whereas B-type *rif* and *Pfmc-2TM*, respectively, were found to be expressed, suggesting that the latter two gene families may encode proteins that are not targeted to the erythrocyte surface and therefore do not participate in endothelial receptor binding. Indeed, surface exposition of PfEMP-1 and recently of STEVOR has been already demonstrated [6], [21] and various studies imply that A-type RIFIN proteins are transported via the Maurer's clefts to the erythrocyte membrane [16], [19], [45]. Moreover, PfEMP-1, RIFIN and STEVOR proteins were previously determined as targets of the adaptive antibody-mediated immunity against *P. falciparum* malaria [6], [46], [47], suggesting exposure of these proteins at the outside of the erythrocyte membrane bilayer, where they are accessible to serum antibodies. Our finding that B-type RIFIN and PFMC-2TM proteins may not be surface exposed is in agreement with the previously determined intracellular localization of B-type RIFIN proteins [16]. Alternatively, as has been predicted for PfMC-2TM proteins, the molecules may contain rather small protein domains of only a few amino acid residues, which are exposed to the erythrocyte surface [13].

The *ex vivo* and subsequent *in vitro* analysis of IE presented here, indicates a correlation between transcription of particular parasite multicopy gene families and binding of IE to endothelial receptors. Whether all of the various gene products participate in receptor binding or only a subset, remains to be determined. Moreover, the mechanism that is responsible for the activation of multicopy gene family expression after a period of four weeks of parasite *in vitro* cultivation is presently unknown. However, MSP-1 genotyping clearly indicates that this late expression of multicopy gene families was not the result of outgrow of an initially underrepresented parasite genotype.

Of course, studies analyzing clinical parasite isolates have certain limitations, in particular time course experiments can only be performed once, cannot be repeated and the amount of material is usually rather low. Therefore, we could not perform the static adherence assays and transcription analysis at all time points simultaneously. Nevertheless, we found a strong association between expression of particular multicopy gene families and cytoadhesion of infected erythrocytes. However, analyses of multicopy gene families are restricted to the primer pairs applied, which might not cover all gene variants of the variant surface protein families and therefore might generate biased amplicons. For this reason, the primer pairs used were validated with genomic DNA from 3D7 as well as from the parasite isolate of the splenectomized patient (Fig. S1, Fig. S2). As both DNA templates revealed similar results of large proportions of amplified genes, it is reasonable to assume that the lack of expression of the various multicopy gene families in the *ex vivo* sample as well as during the first 12 days of culture was not the result of biased amplification. Unfortunately, the *var* primers used in this study do not recognize the *var2csa* gene responsible for pregnancy-associated malaria. Therefore, the expression of this unique gene cannot be excluded but is very unlikely in a multi-gravid non-pregnant woman.

Questions remain why parasitemia increased substantially from an extremely low level to a very high level after removal of the spleen. Similar observations have been reported from other HMS patients following splenectomy [38]–[40]. The patient presented here is considered semi-immune as she has been living in an area hyperendemic for malaria for about 50 years and repeated exposure to malaria parasites is indicated by the massive deposition of hemozoin in the spleen and high antibody titers against *P. falciparum* antigens. The acquired broad antibody repertoire to virtually every parasite protein expressed on IE surfaces may have resulted on the one hand in inhibition of sequestration and on the other hand in sensitization of IE. Both lack of sequestration and sensitization will result in accelerated clearance of IE by the spleen. Thus, a selection may have taken place towards parasites that do not express molecules on the erythrocyte surface. However, these IE are also unable to sequester and therefore only a very small proportion of IE is able to survive which by-pass the spleen. Following splenectomy this specific parasite population has expanded to high density.

To our knowledge this is the first report on the expression of *P. falciparum* multicopy gene families in correlation with binding properties of *ex vivo* parasites from a semi-immune malaria patient following splenectomy. To prove or disprove the hypothesis of a selection of parasites in semi-immune individuals that do not expose proteins on the erythrocyte surface, further parasite isolates have to be investigated. However, these investigations have to be performed early after IE have been taken from the patient. As shown in our example, the parasites may regain expression of surface molecules and binding to endothelial receptors within a short period of *in vitro* culture.

## Supporting Information

Figure S1 Validation of the various sets of primers using from 3D7. PCR products were ligated into the cloning vector pCR2.1 and a representative number of clones were sequenced. The different pie charts demonstrate the relative abundance of sequences present in the various amplicons. * Pseudogene; ** upsD-type var gene; *** upsB-type var gene; **** amplified sequence identical for the genes PFA0045c/PFA0050c/PFI0035c; ***** amplified sequence identical for the genes PF13_0004/PF11_0009; ****** amplified sequence identical for the genes PF11_0010/PFF1560c/PFC0040w; ******* amplified sequence identical for the genes MAL7P1.215/PF11_0020.
(1.19 MB TIF)

Figure S2 Validation of the various sets of primers using genomic DNA from the parasite isolate of the splenectomized patient. PCR products were ligated into the cloning vector pCR2.1 and individual clones were sequenced. The different pie charts demonstrate the relative abundance of sequences present in the various amplicons. *Sequences identified in RT-PCR transcription analysis of time course experiment.
(1.24 MB TIF)
