# Supplementary figures and images for: Absence of Erythrocyte Sequestration and Lack of Multicopy Gene Family Expression in Plasmodium falciparum from a Splenectomized Malaria Patient
Source: PLoS One. 2009 Oct 14;4(10):e7459. doi: 10.1371/journal.pone.0007459 (PMC2758591; doi:10.1371/journal.pone.0007459)

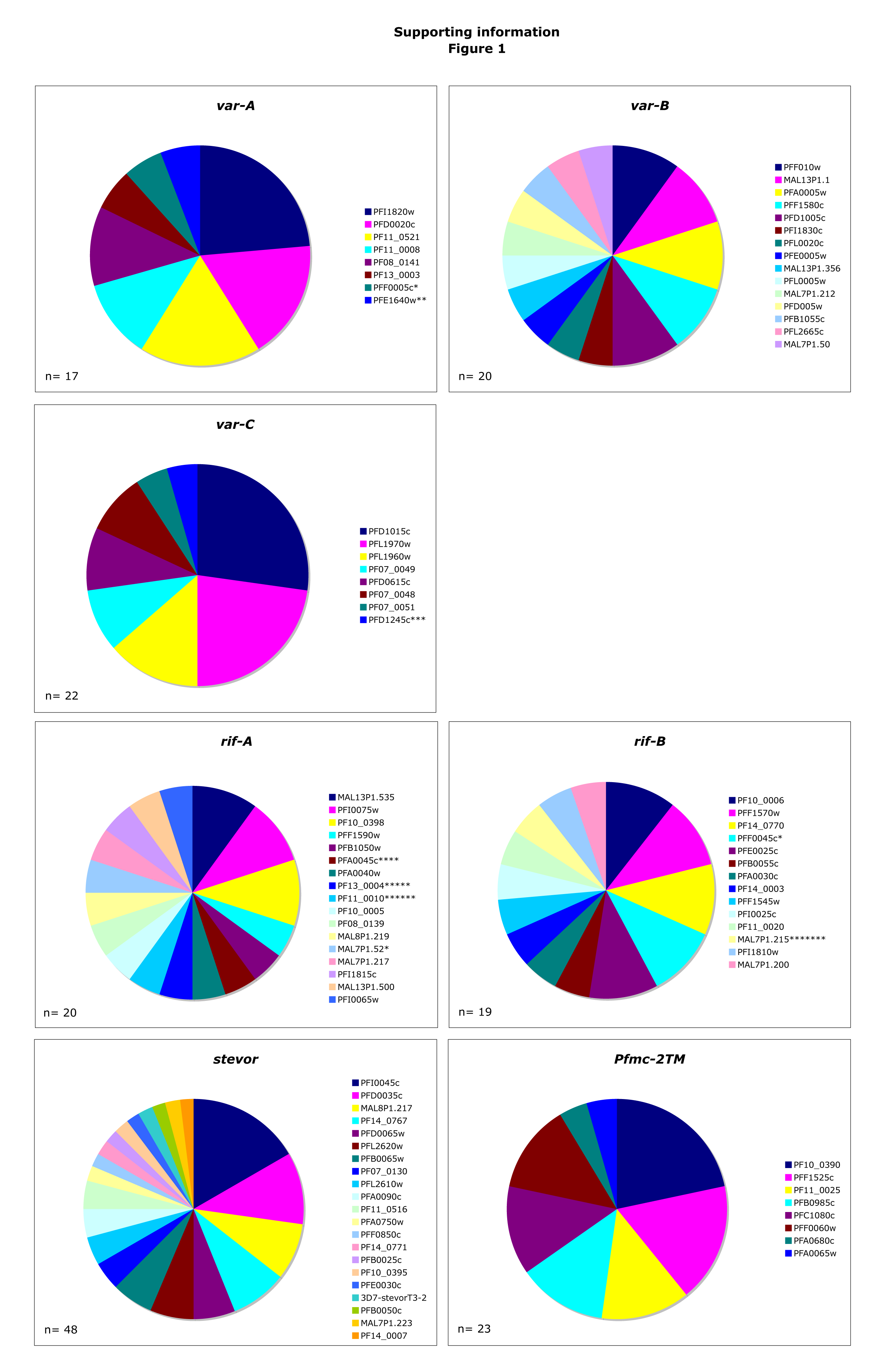

Supplement: Figure S1 — Validation of the various sets of primers using from 3D7. PCR products were ligated into the cloning vector pCR2.1 and a representative number of clones were sequenced. The different pie charts demonstrate the relative abundance of sequences present in the various amplicons. * Pseudogene; ** upsD-type var gene; *** upsB-type var gene; **** amplified sequence identical for the genes PFA0045c/PFA0050c/PFI0035c; ***** amplified sequence identical for the genes PF13_0004/PF11_0009; ****** amplified sequence identical for the genes PF11_0010/PFF1560c/PFC0040w; ******* amplified sequence identical for the genes MAL7P1.215/PF11_0020. (1.19 MB TIF) [file pone.0007459.s001.tif]

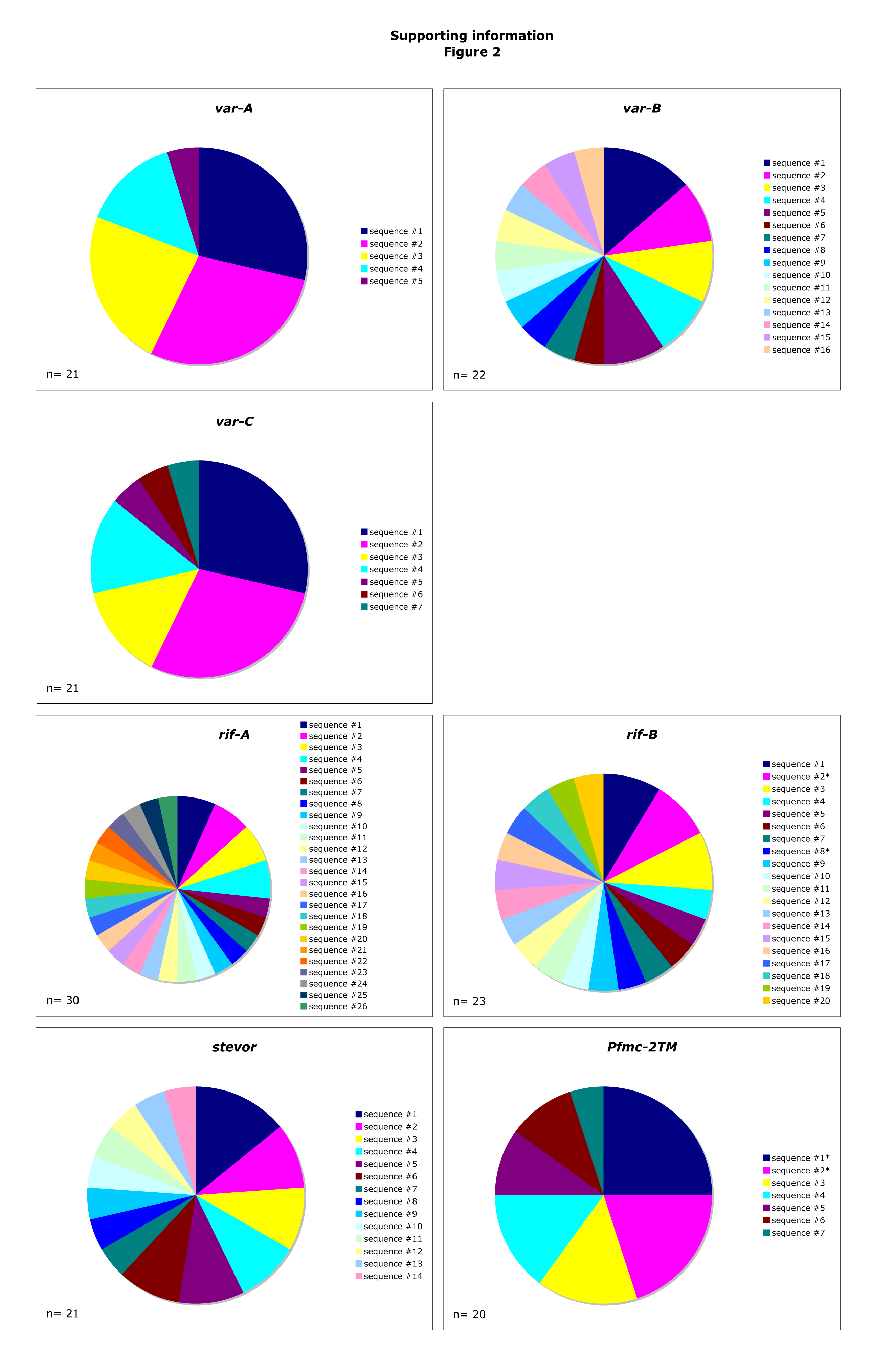

Supplement: Figure S2 — Validation of the various sets of primers using genomic DNA from the parasite isolate of the splenectomized patient. PCR products were ligated into the cloning vector pCR2.1 and individual clones were sequenced. The different pie charts demonstrate the relative abundance of sequences present in the various amplicons. *Sequences identified in RT-PCR transcription analysis of time course experiment. (1.24 MB TIF) [file pone.0007459.s002.tif]
